# A comparison of the breast milk microbiota from women diagnosed with gestational diabetes mellitus and women without gestational diabetes mellitus

**DOI:** 10.1186/s12884-024-06604-x

**Published:** 2024-06-07

**Authors:** Louise Søndergaard Rold, Johan Mikkel Guldbæk, Caroline Steenberg Lindegaard, Stine Kirk, Line Damkjær Nygaard, Caspar Bundgaard-Nielsen, Julie Niemann Holm-Jacobsen, Peter Leutscher, Anne-Cathrine Finnemann Viuff, Søren Hagstrøm, Suzette Sørensen

**Affiliations:** 1 Centre for Clinical Research, North Denmark Regional Hospital, Hjørring, Denmark; 2 Department of Clinical Medicine, Aalborg University, Aalborg, Denmark; 3 Steno Diabetes Center North Denmark, Aalborg, Denmark; 4 Department of Pediatrics and Adolescents, Aalborg University Hospital, Aalborg, Denmark

**Keywords:** Breast milk, Microbiota, Gestational diabetes mellitus, Pregnancy, Pediatrics, Colonization, Infant

## Abstract

**Background:**

Human breast milk (HBM) is a contributing factor in modulating the infant’s gut microbiota, as it contains bacteria that are directly transferred to the infant during breastfeeding. It has been shown that children of women diagnosed with gestational diabetes mellitus (GDM) have a different gut microbiota compared to children of women without GDM. Our hypothesis is therefore that women with GDM have a different HBM microbiota, which may influence the metabolic function and capacity of the child later in life. The aim of this study was to investigate whether women with GDM have a different breast milk microbiota 1–3 weeks postpartum compared to women without GDM.

**Methods:**

In this case-control study, a total of 45 women were included: 18 women with GDM and 27 women without GDM. A milk sample was collected from each participant 1 to 3 weeks postpartum and the bacterial composition was examined by 16 S rRNA gene sequencing targeting the V4 region.

**Results:**

High relative abundances of *Streptococcus* and *Staphylococcus* were present in samples from both women with and without GDM. No difference could be seen in either alpha diversity, beta diversity, or specific taxa between groups.

**Conclusion:**

Our results did not support the existence of a GDM-associated breast milk microbiota at 1–3 weeks postpartum. Further research is needed to fully understand the development of the gut microbiota of infants born to mothers with GDM.

**Supplementary Information:**

The online version contains supplementary material available at 10.1186/s12884-024-06604-x.

## Introduction

Gestational diabetes mellitus (GDM) is defined as impaired glucose tolerance detected during pregnancy. Worldwide, the prevalence of GDM is increasing and affecting up to 14% of pregnant women [1]. Known risk factors for developing GDM include a family history of type 2 diabetes mellitus, previous delivery of a macrosomic infant (> 4500 g), pre-pregnancy BMI > 27 kg/m², glucosuria, and polycystic ovarian syndrome [2]. GDM affects both mother and child with complications such as preeclampsia, neonatal hypoglycemia, and congenital malformations [3–5]. Additionally, GDM predisposes both mother and child to diabetes, obesity, and cardiovascular disease [6–11]. The increased risk of diabetes, obesity, and cardiovascular disease in children born to mothers with GDM is not fully understood, and emerging research suggests an involvement of the microbiota.

The human gut microbiota is complex and comprises numerous microorganisms each playing a crucial role in maintaining normal physiological functions [12]; some of these may cause increased weight gain, while others can induce insulin resistance [13–15]. Disruption of the microbiota, known as dysbiosis, has been observed in individuals with obesity, pre-eclampsia, type 2 diabetes, and GDM [13, 16–19]. The role of the microbiota in disease development is further supported by fecal microbiota transplantation (FMT) studies in animals, where a dysbiotic obesity-associated microbiota can induce increased body fat and insulin resistance in the recipient [15, 20]. Furthermore, studies have shown an association between infant gut dysbiosis and the development of type 1 diabetes, obesity, and asthma [21, 22], which emphasizes the potential early-life influence of the gut microbiota on disease development later in life. Children born to women with GDM have a distinct gut microbiota at birth, 2 weeks, and 5 years postpartum [23–25]. This suggests maternal transmission of a GDM-associated microbiota, which may have a modulating effect on the child’s gut colonization and disease development later in life compared to children born to mothers without GDM.

The process of gut colonization begins early in life and is initially dominated by *Bifidobacterium* before transitioning into a more diverse and stable gut microbiota by the age of three years [26]. The early presence of *Bifidobacterium* is important for normal gut microbiota establishment, as it has beneficial effects on the host such as immune system modulation and defense against pathogens [27]. The colonization of bacteria early in life [28] is influenced by gestational age at birth, mode of delivery, maternal health, and feeding with breast milk or formula [26, 29]. During the first months of life, breast milk is crucial in modulating the infant’s gut microbiota [30–32]. Bacterial genera from breast milk have been identified in infant stools, indicating a horizontal maternal transfer of bacteria [32]. Through this process, the mother seemingly provides the infant with microbial agents that colonize the gastrointestinal tract and contribute to the establishment of the infant gut microbiota [32]. The breast milk microbiota is mainly dominated by *Staphylococcus* and *Streptococcus*, but also contains *Serratia, Pseudomonas, Corynebacterium, Ralstonia, Propionibacterium, Finegoldia, Sphingomonas, and Bifidobacterium* [33–37]. In addition to the bacteria themselves, breastmilk is also known to contain prebiotic components such as human milk oligosaccharides (HMOs), which may also contribute to modulation of the infant’s gut microbiota [38]. Maternal factors such as pre-pregnancy BMI [36], weight gain during pregnancy [36], lactation state [34], consumption of antibiotics [39, 40], maternal secretor status [41], and mode of delivery [42] may influence breast milk composition. In addition, disease during pregnancy may also be an important factor. For instance, a few previous studies have compared breast milk microbiota from women who had GDM with that of former healthy pregnant women, although with conflicting results, as one study identified specific bacterial differences [39] while the other found no distinctions [43]. Notably, these studies assessed different time points; one observed variations in colostrum samples [39], while another found no differences in breast milk at 3 months postpartum [43]. Consequently, further investigation is needed to clarify whether and when differences exist in breast milk microbiota between women with and without GDM.

The aim of this study was to investigate whether women with GDM have a different breast milk microbiota 1–3 weeks postpartum compared to women without GDM.

## Methods

### Study participants

Participants were recruited from the maternity wards of the North Denmark Regional Hospital, Denmark, and Aalborg University Hospital, Denmark, shortly after delivery (1–3 days). Inclusion criteria were women whose gestational age at delivery was 37 + 0 to 41 + 6 weeks, who delivered a healthy child. Women were excluded if they had type 1 or type 2 diabetes, if they were treated with antidepressants, or if they had pre-eclampsia. In total, 18 women with GDM and 27 women without GDM were included.

### Anthropometric and clinical data

The women filled out a questionnaire regarding their use of antibiotics within the last three months, height, pre-pregnancy weight, GDM diagnosis, insulin treatment, delivery mode, and time of sample collection. The diagnostic criterion for GDM was based on a 2-hour 75 g oral glucose tolerance test with a 2-hour glucose ≥ 9.0 mmol/L.

### Sample collection

Breast milk samples were collected by the mothers 1–3 weeks after birth. The women collected 10–20 ml of milk from one of the breasts. Before sample collection, the breast was cleaned with disinfection wipes. The women were asked not to nurse from the chosen breast three hours prior to sample collection. The breast milk sample was collected manually by massaging the breast. The women were instructed to wear sterile gloves and to discard the first few drops of milk to reduce contamination from the mother’s skin. The breast milk sample was collected into a sterile tube and immediately stored at -20˚C at the homes of the participants for up to 3 days and were subsequently transported on ice to the research laboratory at the North Denmark Regional Hospital, Denmark, where they were transferred to a -80 °C freezer and stored until further processing.

### DNA extraction and 16 S rRNA gene sequencing

Total DNA was extracted from 1.5 mL breast milk. Initially, samples were centrifuged at 13,000 x g at 4 °C for 20 min. The fat layer and supernatant were removed without disturbing the pellet. DNA from the pellet was isolated using the QIAamp PowerFecal Pro DNA kit (Qiagen) automated on a QIAcube® (Qiagen) according to the manufacturer’s protocol. The total concentration of DNA was measured using the Qubit dsDNA HS Assay kit (Thermo Fisher Scientific). Duplicate DNA extractions were performed for all participants.

DNA was subjected to 16 S rRNA gene sequencing by DNAsense (Denmark), using a modified version of an Illumina protocol [44], as previously described [45]. Briefly, the extracted DNA was used as a template for PCR amplification of the V4 region of the 16 S rRNA gene, using the following standard primers (515 F (Parada) GTGYCAGCMGCCGCGGTAA and 806R (Apprill) GGACTACNVGGGTWTCTAAT) [46] and 30 amplification cycles. The resulting amplicon libraries were subsequently amplified in a second PCR using 8 cycles, and sequenced using a MiSeq (Illumina, USA), with the MiSeq Reagent Kit v3 (Illumina). A 10% PhiX contol library (Illumina) was spiked in to overcome low complexity issues.

### Bioinformatics

After sequencing, the USEARCH v11 Amplicon Analysis pipeline [47]was applied to remove PhiX sequences and to demultiplex the samples. The resulting sequences were imported into the QIIME2 v 2022.2 platform. Forward reads were filtered for primers, truncated to 264 base pairs, and denoised using Deblur with standard parameters. The resulting ASVs were aligned with MAFFT through q2-alignment which was used for the construction of a phylogenetic tree using FastTree 2. The Naïve Bayesian classifier, which is implemented in the q2-feature-classifier, was trained against the SILVA 138 SSU reference database and used to assign taxonomy to each ASV. Feature tables were then exported to R v 4.3.2 for downstream analyses [48]. As mentioned, DNA extraction was performed twice for each sample, which resulted in two 16 S rRNA sequencing results (replicate 1 and 2) from each participant. Both replicates were used for all analyses.

Potential contaminating bacteria were identified and removed using the Decontam package v 1.22.0. We used the combined method (threshold of *P* = 0.1) that both utilizes the frequency of ASVs in negative controls compared to samples, as well as checks for correlation between initial DNA quantity compared to the relative abundance of ASVs in samples. Alpha- and beta-diversity were investigated using the packages phyloseq v 1.46.0 (https://github.com/joey711/phyloseq) and Ampvis2 v 2.8.6. Alpha diversity metrics included ASV richness, Shannon diversity index, and Faith’s phylogenetic diversity. Differences in alpha diversity metrics between groups, were analyzed using linear mixed-effect models, with participant ID as random effect, to adjust for the variability introduced by sample replicates. Variations in beta diversity were determined using weighted UniFrac, unweighted UniFrac, and Bray Curtis dissimilarity. Differences in beta diversity were analyzed using PERMANOVA as implemented in the Vegan package, with 999 permutations and participant ID added as strata, to adjust for the effects of replicates, while dispersion was tested using Betadisper. The resulting variations in beta-diversity were visualized using principal coordinate analysis (PCoA) with ellipses depicting 95% confidence intervals. The weighted UniFrac distance between samples was subsequently used for Agglomerative Hierarchical Clustering, using Ward’s minimum variance method, as implemented in the hclust command in R [49]. The number of clusters was subsequently selected based on the Silhouette clustering metric, based on potential clusters between 2 and 6. Finally, a dendrogram was used to visualize the clustering, using the Dendextend package v. 1.17.1 in R [50]. The relative abundance of bacteria in the samples corresponding to the individual clusters, was visualized using stacked barplots with phylum and genera, using the Microshades package v. 1.11 in R [51]. The analysis of compositions of microbiomes with bias correction (ANCOM-BC) was used to investigate differentially abundant taxa between women with and without GDM, at genus level, using the ancombc2 command in the ANCOMBC package v. 2.4.0, with pseudo_sens set to true. Patient ID was used as random effects, to adjust for the variability introduced by replicates. Taxa were removed from the ANCOM-BC2 analysis if they were present in less than 10% of sample. Only genera that were consistently associated with either GDM or non-GDM, even after pseudo-count addition (passed sensitivity analysis), were considered truly significant. P-values were adjusted for multiple comparisons using the Benjamini-Hochberg approach, where an adjusted p-value < 0.05 was considered significant for all analyses.

### Statistics

All statistical analyses were performed in R v 4.3.2 [48]. Mean and median values are stated as mean ± SD and median (Q1-Q3), respectively. For continuous, clinical data, (BMI and sample collection time) distribution and variance were determined using Shapiro-Wilks test and Bartlett’s test, respectively. Depending on the results, differences between clinical groups for continuous clinical data were analyzed using Student’s t-test on normally distributed data with equal variance, while non-normal distributed data were analyzed using the Wilcoxon rank-sum test. Fisher’s exact test was used to analyze differences in categorical data. The p-values were adjusted for multiple hypothesis testing, using the Benjamini-Hochberg approach. A multiple-testing adjusted p-value < 0.05 was considered significant.

## Results

### Characteristics of study participants

In total, 45 participants were included in this study (Table 1). These were 18 women with GDM and 27 women without GDM. No statistical difference could be observed in sample collection time (days after delivery) (*p* = 0.80), pre-pregnancy BMI (*p* = 0.40), and antibiotic treatment in the pregnancy and during delivery (*p* = 0.07) between the groups. However, a significant difference could be seen in mode of delivery (*p* = 0.002), insulin treatment (*p* = 0.008) and previous GDM diagnosis (*p* = 0.02).

**Table 1:** Characteristics of study participants. A total of 45 women were included in the study. However, some data were not available for all women. This included mode of delivery (*n* = 40), antibiotic treatment (*n* = 38), and insulin treatment (*n* = 44)

Characteristic	GDM, *n* = 18^*1*^	Non-GDM, *n* = 27^*1*^	*p*-value^2^
**Days after delivery**	10.5 (9.3, 13.8)	11.0 (7.5, 15.5)	0.80
**Pre-pregnancy BMI**	27.0 (22.5, 31.9)	24.2 (21.6, 30.3)	0.4
**Pre-pregnancy BMI group**			> 0.90
< 25	8/18 (44,4%)	14/27 (51,9%)	
25–30	3/18 (16,7%)	5/27 (18,5%)	
> 30	7/18 (38,9%)	8/27 (29,6%)	
**Mode of delivery**			0.002*
C-section	6/16 (37,5%)	0/24 (0%)	
Vaginal birth	10/16 (62,5%)	24/24 (100%)	
**Antibiotic treatment**	4/15 (26,7%)	1/23 (4.3%)	0.07
**Insulin treatment**	5/18 (27,8%)	0/26 (0%)	0.008*
**Previous GDM**	4/18 (22,2%)	0/27 (0%)	0.02*

^*1*^ Median (IQR); n (%) ^*2*^ Wilcoxon rank sum test for continuous data; Fisher’s exact test for categorical data

### Quality control and sequencing data

A total of 87 breast milk samples and 10 negative controls gave rise to 1,498,087 reads, with an average of 15,444.2 reads per sample (See Supplementary material 1). Taxonomic assignment led to identification of 566 unique ASVs. Removal of contaminants using the Decontam package, led to removal of five ASV (see Supplementary material 2). Duplicates were avaible for all but three study participants (MM_405, MM_511, and MM_64), and were observed to be highly similar (see Supplementary material 1). After Decontam analyses and removal of negative controls, we ended up with 87 samples, containing a total of 540 ASVs with taxonomy assigned for 99.81%, 99.26%, and 95.00% at phylum, family, and genus levels, respectively.

### No difference in neither alpha diversity nor beta diversity in breast milk samples from women with and without GDM

**Fig. 1:**
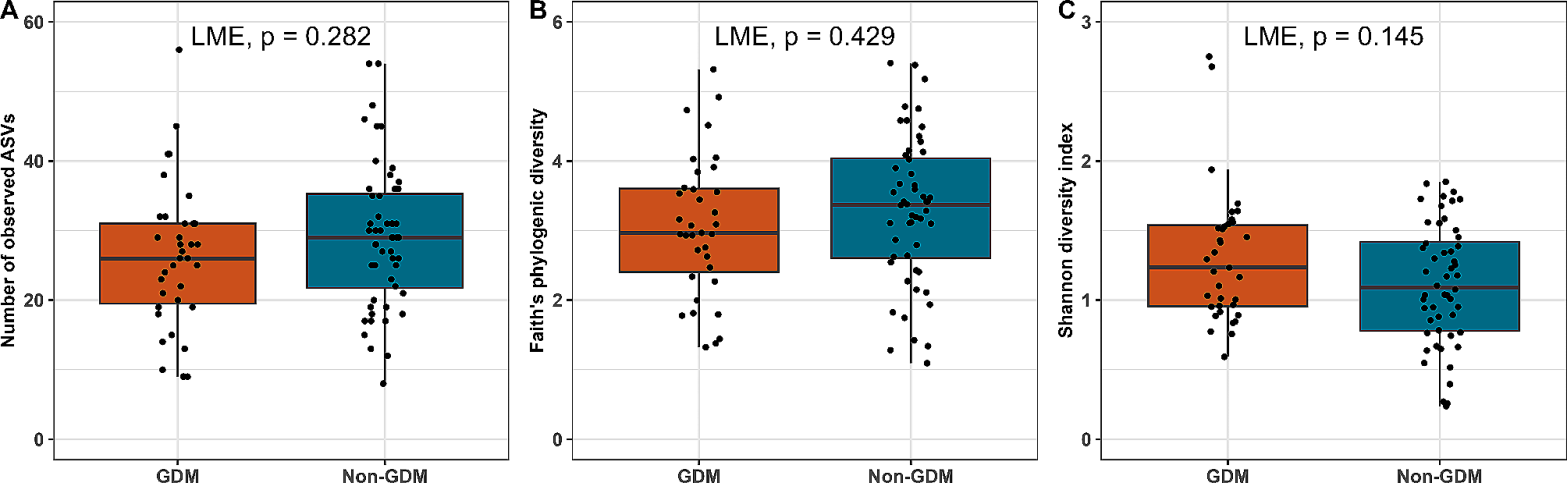
Differences in alpha-diversity of breast milk samples from women with or without GDM. Alpha-diversities were analyzed using **a**) ASV richness, **B**) Faith’s phylogenic Diversity, and **C**) Shannon diversity index

To investigate bacterial alpha-diversity we calculated ASV richness, Faith’s phylogenetic diversity, and Shannon diversity index (Fig. 1). No significant difference could be observed between women with and without GDM in either of the alpha diversity measures.

**Fig. 2:**
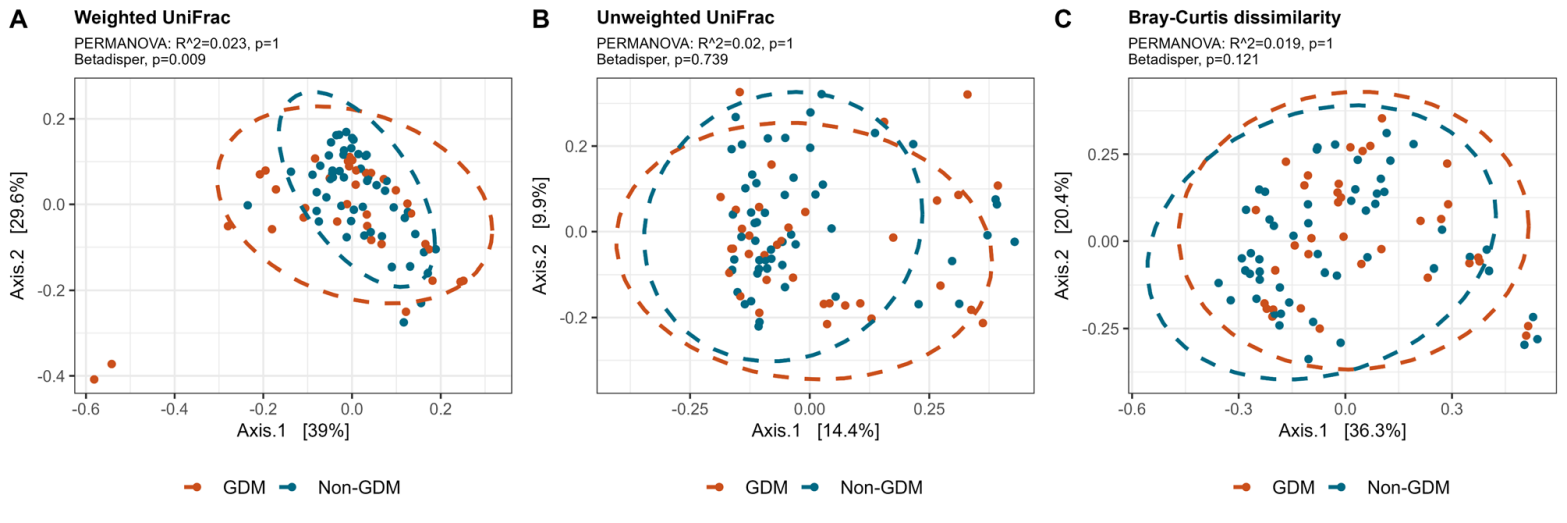
Beta-diversity variations in breast milk microbiota for women with or without GDM. The beta-diversity is presented using principal coordinate analysis (PCoA), with either **A**) weighted UniFrac, **B**) unweighted UniFrac, or **C**) Bray-Curtis dissimilarity. Ellipses depict 95% confidence intervals. PERMANOVA results are indicated with adjusted p-values and R2 and represent the overall differences in beta-diversity, while differences in dispersion were tested using betadisper and indicated using p-values

To assess potential differences in bacterial communities between study groups, we investigated the beta diversity of the breast milk microbiota using PCoA, and tested variance by PERMANOVA. No difference could be seen between women with and without GDM in beta diversity for Weighted UniFrac, Unweighted UniFrac, and Bray Curtis dissimilarity (Fig. 2).

**Fig. 3:**
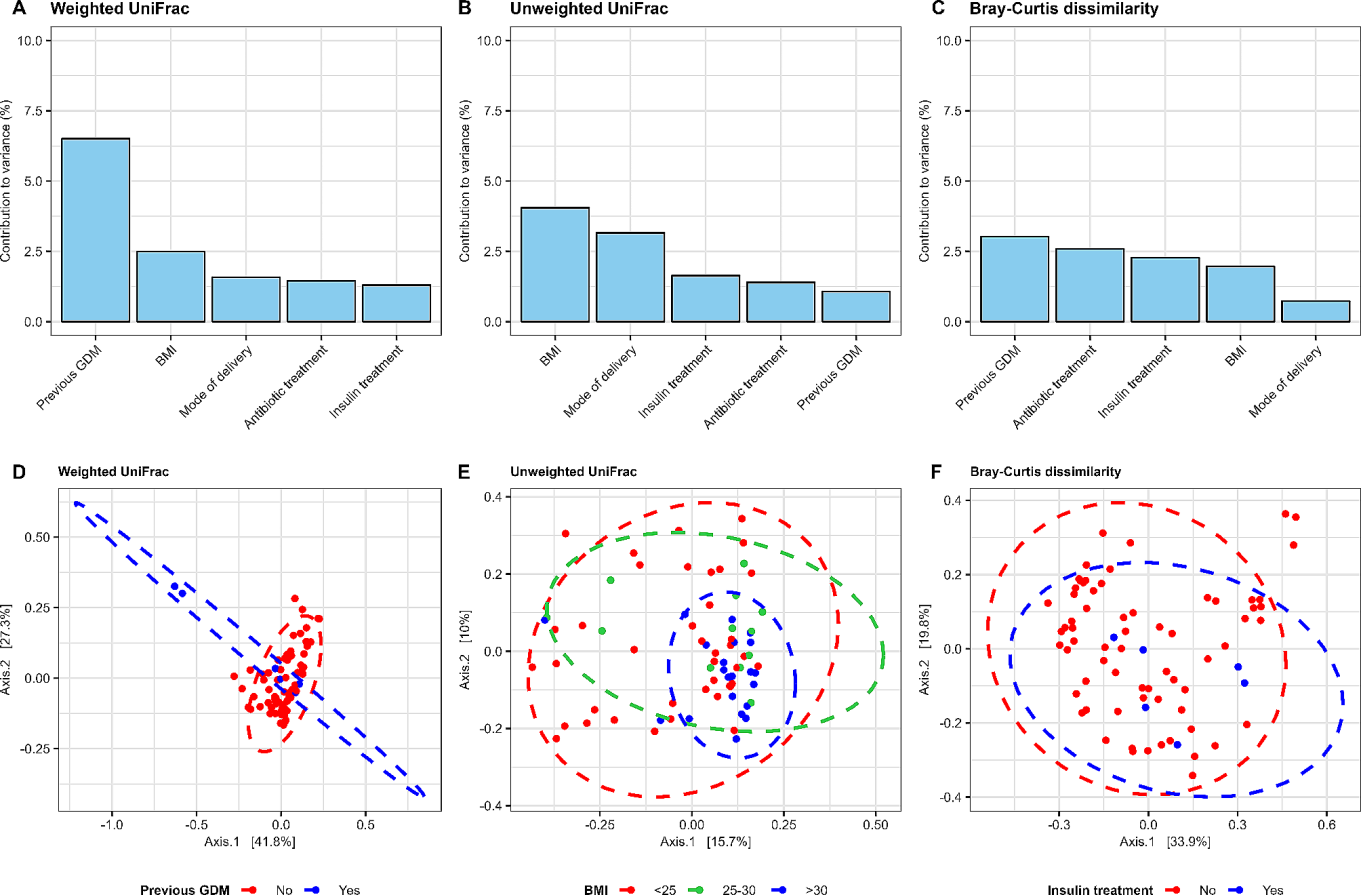
Explained variance of 5 covariates. The bar plots show the amount of variance (r^2^) explained by each covariate. **A**) illustrates the variance of each covariate when using weighted UniFrac, **b**) illustrates the variance of each covariate when using unweighted UniFrac, **c**) illustrates the variance of each covariate when using Bray Curtis dissimilarity, d-f) show PCoA plots for the covariate that explains most in the data set for weighted UniFrac, unweighted UniFrac and Bray Curtis dissimilarity respectively

Potential confounders were added as fixed effects to a subsequent PERMANOVA model, including BMI group, insulin treatment, antibiotic treatment, mode of delivery and if the women had a previous GDM diagnosis. None of the factors had a significant effect on the microbiota composition, though previous GDM diagnosis and BMI were shown to have the largest effect on the data set (Fig. 3).

### Breast milk microbiota in women with and without GDM is dominated by *Staphylococcus* and *Streptococcus*

**Fig. 4:**
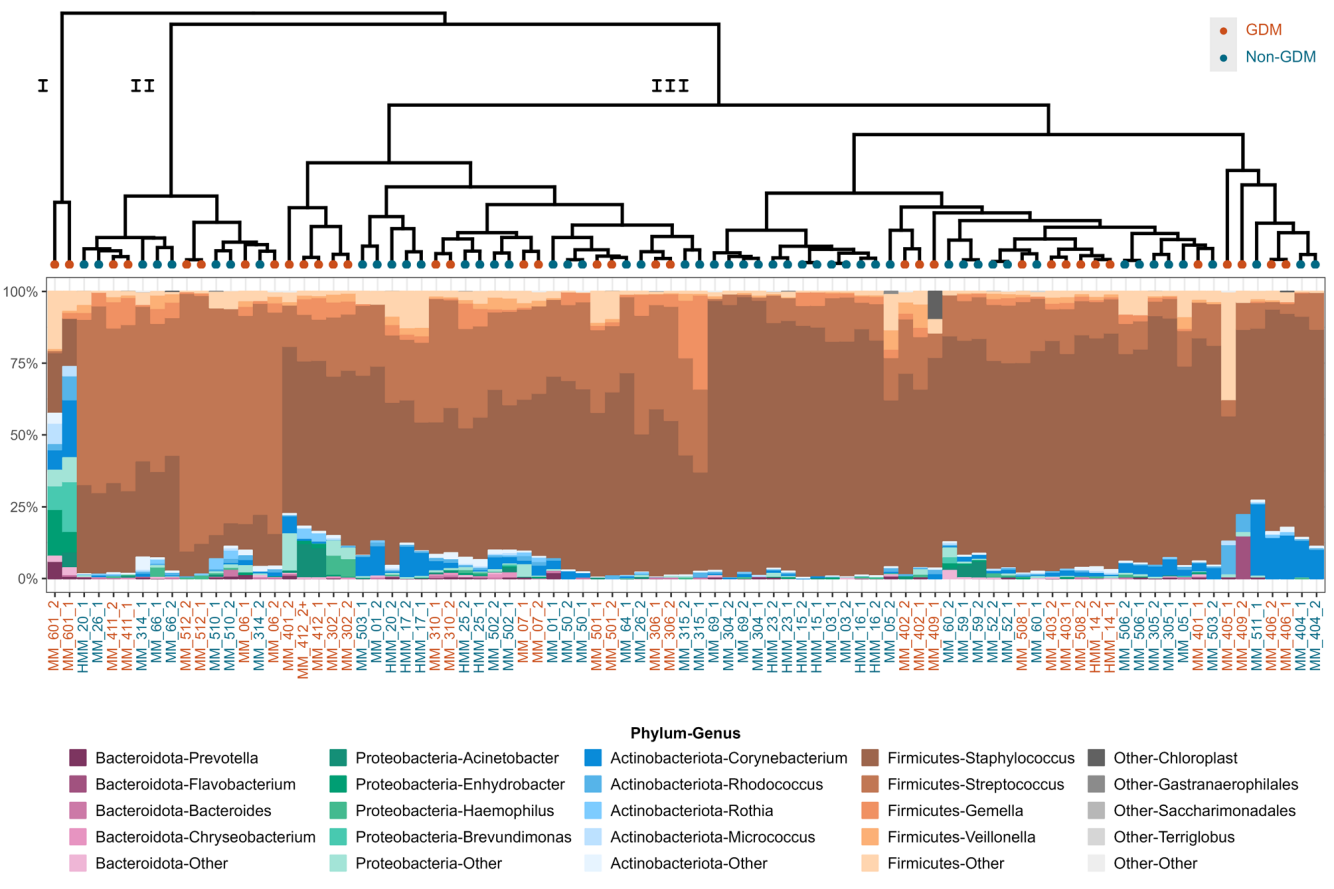
Unsupervised clustering and bacterial composition of samples at the individual clusters. Hierarchical clustering (top) was performed using Ward’s minimum variance method, on weighted UniFrac distance between the individual breastmilk samples. The Roman numerals represent the three clusters. The number of clusters chosen was based on the Silhouette metric. The stacked bar plots represent the relative abundance of breast milk microbiota for each sample in each cluster, using the taxonomic classifications phyla and genera. The ID of each participant is colored based on GDM status

Breast milk samples both from women with and without GDM were dominated by the phylum Firmicutes with a small contribution from phylum Actinobacteria and phylum Proteobacteria (Fig. 4). For genera, we observed that all samples were dominated by *Staphylococcus* and *Streptococcus* (Fig. 4).

Hierachial clustering was subsequently performed based on weighted UniFrac, which demonstrated the presence of two major clusters (II and III) that were evenly represented by both women with and without GDM (Fig. 4). Cluster II had a higher *Streptococcus* to *Staphylococcus* ratio compared to cluster III. A single sample (cluster I) was markedly different from the other samples with a higher diversity and almost complete lack of *Streptococcus*.

ANCOM-BC was used to evaluate differential abundance of bacterial genera between women with and without GDM. No significant differences were observed when adjusting for multiple comparisons (Supplementary material 3).

## Discussion

Our study investigated whether women with GDM have a different breast milk microbiota 1–3 weeks postpartum compared to women without GDM. We did not observe significant differences in the composition of breast milk microbiota between the two groups, concerning either alpha diversity, beta diversity or specific taxa. However, our study found that the dominant phylum in breast milk samples from both women with and without GDM was Firmicutes. This observation is in line with earlier studies, indicating that Firmicutes is a consistent and predominant phylum in breast milk microbiota across varying populations [33–37]. Furthermore, our analysis at genus level revealed a composition resembling the core breast milk microbiota reported in previous studies. Notably, genera in our samples included *Staphylococcus*, *Streptococcus*, *Corynebacterium*, *Finegoldia*, and *Bifidobacterium*. These findings were in line with our expectations based on the existing literature [33–37].

We expected that women with GDM would have a different breast milk microbiota as previous studies have reported a dysbiosis in the gut, vagina, colostrum, and mouth of women with GDM, as well as different gut microbiota in infants of GDM-affected mothers [52–54]. However, our findings did not support this. Furthermore, our study contrasted with the study by Gámez-Valdez et al. [39] who reported a higher relative abundance of *Staphylococcus* and *Prevotella* in breast milk samples from women with GDM compared to women without GDM [39]. The differences in our results and those of Gámez-Valdez et al. [39] may be attributed to the differences in sample collection procedures, the isolation methods, and the analysis pipelines. We used Deblur for sequence processing, which removes sequences, while Gámez-Valdez et al. [39] used DADA2, which attempts to correct sequences [39]. Moreover, Gámez-Valdez et al. employed LEfSE for group comparisons, whereas we used ANCOM-BC2. ANCOM-BC2 accounts for zero inflation, rendering the analysis more stringent. Thus, methodological differences may partly explain the different findings [34]. Another difference between our study and the study by Gámez-Valdez et al. [39] was the sample collection time. Our study analyzed transition/mature milk, while Gámez-Valdez et al. [39] analyzed colostrum. Previous research has suggested that the composition of milk microbiota changes over time [34, 37], and this temporal variation could explain the differences observed in colostrum, which may not necessarily be present in mature milk. To our knowledge, no studies have explored whether breast milk microbiota composition changes over time from a more GDM-associated composition to one resembling that of healthy controls. Another study has investigated breast milk microbiota in women with and without GDM three months postpartum and found no significant differences [43]. This suggests that a GDM-associated breast milk microbiota might only be present in the early stages of lactation, and based on our results is already gone 1–3 weeks postpartum. This was further supported by a study reporting higher glucose levels in colostrum samples compared to samples from mature milk [38]. It would therefore be interesting to follow a larger number of women with and without GDM to see if the microbiome is different in the beginning but not later in the lactation period.

The reason we did not see a difference in the breast milk microbiota between women with and without GDM could also be due to the nature of the samples. Breast milk has a low microbial biomass [37], that is highly dominated by *Streptococcus* and *Staphylococcus*, which could overshadow any minor variations in the breast milk composition. Importantly, these bacteria are believed to originate from the maternal skin and infant oral cavity, opposed to the breast milk itself, suggesting that breast milk does not contain bacteria but is merely contaminated from external areas [55]. Other studies have, however, found bacteria (genus level) in breast milk samples that were not simultaneously found on the mother’s skin or the child’s mouth, pointing towards the existence of a breast milk microbiota [56].

Even though we do not find a GDM-associated breast milk microbiota, it is still possible that the mother indirectly transfers a GDM-associated bacterial profile to the child. For instance, different compositions of HMO´s in breastmilk from women with GDM vs. women without GDM may also explain differences in infant gut microbiota colonization. Several studies have shown that the HMO profile in women with GDM is different from women without GDM [38, 57, 58]. One study showed a lower number of HMOs in women with GDM collected six days after birth and observed a delayed colonization of *Bifidobacterium* and *lactobacillus* in the infant gut [38]. This suggested that the low level of HMOs in breast milk samples from mothers with GDM during early lactation, could be involved in the disruption of normal gut colonization in the infant. Other routes of transmission may also occur, e.g. by the maternal gut and vaginal environments [54, 59]. Differences in infant gut microbiota could therefore be explained by microbial transfers from these maternal environments rather than from breast milk. This was supported by a study demonstrating similarities in gut microbiota composition between the mothers with GDM and their infants [23, 54].

Our study has some limitations. The clinical data were collected through self-reporting questionnaires and some data points are missing. Additionally, our sample size was relatively small. Furthermore, while all the women included in the study initially planned to breastfeed directly, we lack the information on whether some of them transitioned from direct breastfeeding to indirect breastfeeding before the sample collection. Finally, in our analysis, we only examined data at the genus level and not at the species level, and differences at lower taxonomic levels or at functional levels may therefore have been missed [16]. Our study does also have several strengths. Since the breast milk microbiota may change during the lactation period, it is a strength of our study that all breast milk samples were collected within a limited time period. Furthermore, the women were instructed to collect the samples by manually expressing milk from a breast that had not been nursed from for a period of three hours, and the first volume of milk was discarded to reduce skin contamination.

In conclusion, our results did not support the existence of a GDM-associated breast milk microbiota at 1–3 weeks postpartum. However, it is still plausible that breast milk may exert an influential role in shaping the development of a GDM-associated infant gut microbiota via other breast milk components. Further research is therefore needed to fully understand the development of the gut microbiota of infants born to mothers with GDM.

### Electronic supplementary material

Below is the link to the electronic supplementary material.

Supplementary Material 1

Supplementary Material 2

Supplementary Material 3

## Data Availability

The datasets generated and/or analyzed during the current study are available in the NCBI sequence read archive (SRA), available at https://www.ncbi.nlm.nih.gov/bioproject/PRJNA1054972.

## Abbreviations

HBM: Human breast milk
GDM: Gestational diabetes mellitus
FMT: Fecal microbiota transplantation
HMOs: Human milk oligosaccharides
PCoA: Principal coordinate analysis
