# Supplementary material for: A comparison of the breast milk microbiota from women diagnosed with gestational diabetes mellitus and women without gestational diabetes mellitus
Source: BMC Pregnancy Childbirth. 2024 Jun 7;24:412. doi: 10.1186/s12884-024-06604-x (PMC11157733; doi:10.1186/s12884-024-06604-x)
Supplement: Supplementary file 1 — Supplementary Material 1 [file 12884_2024_6604_MOESM1_ESM.docx]

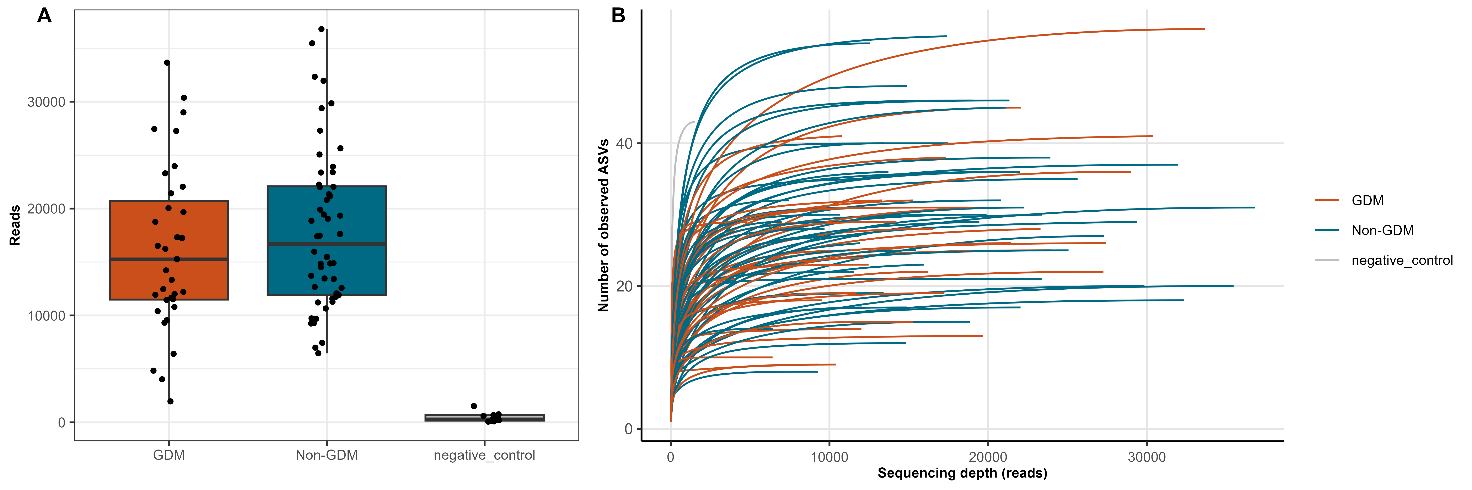


Supplementary figure 1: a) number of reads in each group b) rarefaction curves for all included samples.


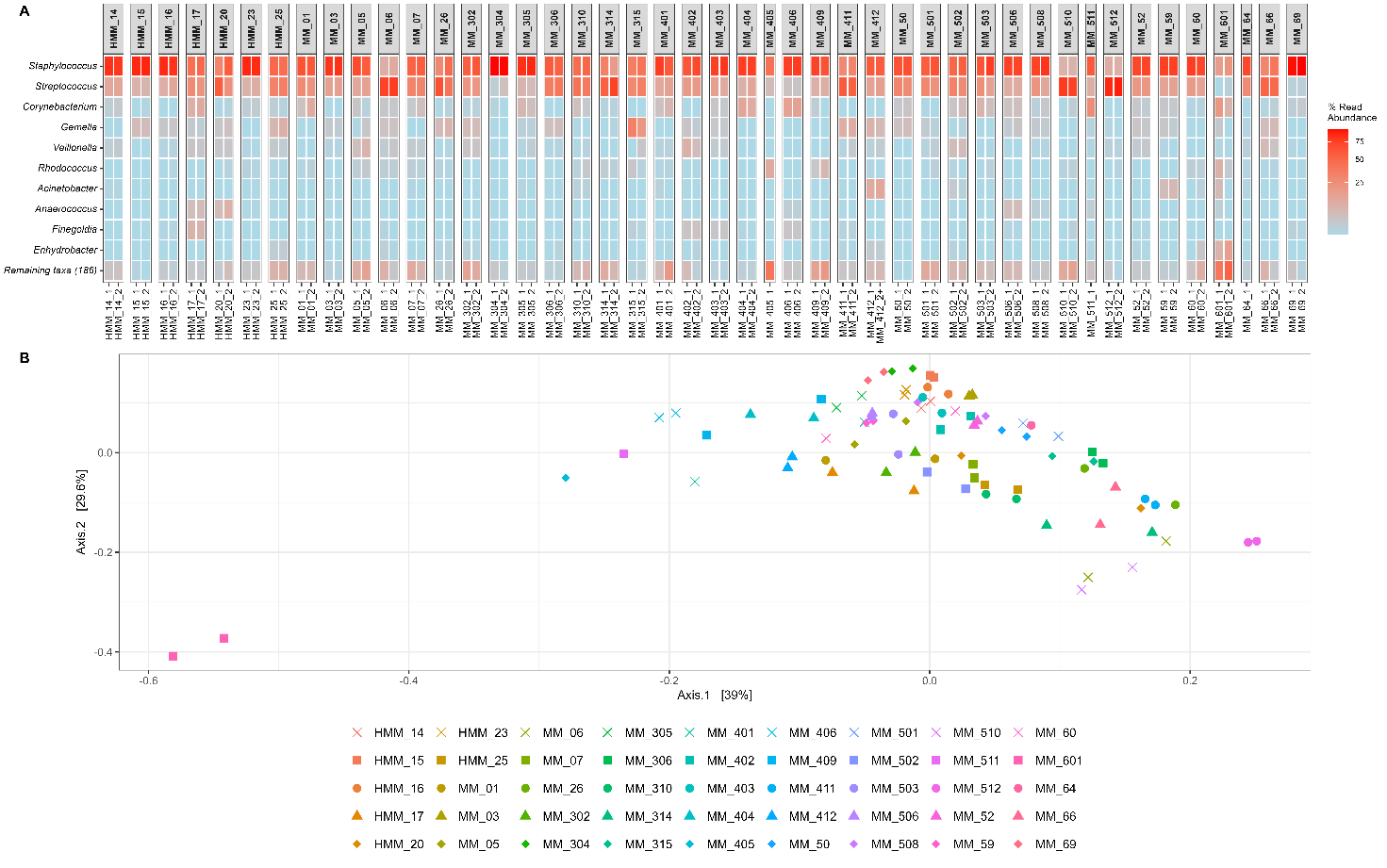


Supplementary figure 2: Comparison of bacterial composition between duplicate samples, visualized as a) a heatmap of relative abundance of bacterial genera in each sample duplicate. b) Principal coordinate analysis (PCoA) with distance calculated using weighted UniFrac. The colors and shapes of dots represent different participants.
